# Evaluation of health-related quality of life of Covid-19 patients: a hospital-based study in South Central Ethiopia

**DOI:** 10.1186/s12955-021-01900-y

**Published:** 2021-12-20

**Authors:** Abdene Weya Kaso, Gebi Agero, Zewdu Hurisa, Taha Kaso, Helen Ali Ewune, Alemayehu Hailu

**Affiliations:** 1 School of Public Health, Dilla University, Dilla, Ethiopia; 2 Department of Public Health, College of Health Science, Arsi University, Assela, Ethiopia; 3 Department of Internal Medicine, College of Health Science, Arsi University, Assela, Ethiopia; 4 Department of Surgery, College of Health Science, Arsi University, Assela, Ethiopia; 5 Bergen Centre for Ethics and Priority Setting, Department of Global Public Health and Primary Care, University of Bergen, Bergen, Norway

**Keywords:** Quality of life, HRQOL, Covid-19, Arsi Zone, Ethiopia

## Abstract

**Background:**

Covid-19 causes a wide range of symptoms in patients, ranging from mild manifestations to severe disease and death. This study assessed the health-related quality of life (HRQOL) and associated factors of Covid-19 patients using primary data from confirmed cases in South Central Ethiopia.

**Methods:**

We employed a facility-based, cross-sectional study design and conducted the study at the Bokoji Hospital Covid-19 treatment centre. A structured questionnaire and the EQ-5D-3L scale were used to collect the data for analysis. The HRQOL results measured by the EQ-5D-3L tool were converted to a health state utility (HSU) using the Zimbabwe tariff. The average health utility index and HSU–visual analogue scale across diverse sociodemographic and clinical characteristics were compared using the Mann–Whitney U test or Kruskal–Wallis test. We employed a multiple linear regression to examine factors associated with HSU values simultaneously. The data were analysed using STATA version 15.

**Results:**

The overall mean HSU score from the EQ-5D was 0.688 (SD: 0.285), and the median was 0.787 (IQR 0.596, 0.833). The mean HSU from the visual analogue scale score was 0.69 (SD: 0.129), with a median of 0.70 (IQR 0.60, 0.80). Those who received dexamethasone and intranasal oxygen supplement, those with comorbidity, those older than 55 years and those with a hospital stay of more than 15 days had significantly lower HSU scores than their counterparts (*p* < .001).

**Conclusion:**

Covid-19 substantially impaired the HRQOL of patients in Ethiopia, especially among elderly patients and those with comorbidity. Therefore, clinical follow-up and psychological treatment should be encouraged for these groups. Moreover, the health utility values from this study can be used to evaluate quality adjusted life years for future cost-effectiveness analyses of prevention and treatment interventions against Covid-19.

## Introduction

Coronavirus disease 2019 (Covid-19) is first discovered in China’s Wuhan Province in December 2019. According to the World Health Organization (WHO) (April 20, 2021), more than 140 million cases and over 3 million deaths have been globally attributed to Covid-19 [1]. In Ethiopia, the first cases of Covid-19 were reported on March 13, 2020. An Ethiopian Ministry of Health report states that more than 240,000 cases and 3,370 deaths have been reported [1]. The pandemic is causing a broad range of health, social and economic crises at a macro and micro level [2].

Covid-19’s wide spectrum of symptoms ranges from mild manifestations to severe disease and death, and some people may have the disease without developing symptoms. The most common symptoms are upper respiratory tract conditions (sore throat, cold symptoms, mild cough), muscle pain and generally feeling unwell. Stomach pains and diarrhoea may occur in some cases, and the loss of the senses of taste and smell is also reported. Some patients may develop pneumonia with severe breathing difficulties, cough and fever and may need to be admitted to intensive care treatment units. Examination of the lungs usually finds changes consistent with viral pneumonia. Death is common among older people, particularly among the elderly with underlying diseases, but death can also occur among people without known risk factors [3, 4].

Health-related quality of life (HRQOL), an essential health care indicator for any disease type [5], measures patients’ overall wellbeing in physical, mental and emotional aspects at a specific time. It can be used in evaluating the severity of a disease, treatment outcomes, patient satisfaction with care, quality of services, overall patient wellbeing and the cost-utility of interventions targeting the disease [5–8]. As Covid-19 is a new disease, however, little is known about its impact on HRQOL. In Italy, a retrospective analysis of HRQOL using SF-36 and involving 673 cases one month after discharge from San Salvatore Hospital in Pesaro found that Covid-19 caused a substantial reduction in patients’ physical and mental health conditions. That study indicates that physical and emotional roles, vitality and social functioning were highly affected dimensions [9]. A retrospective study in China indicates that Covid-19 has a substantial impact on the physical and psychological dimensions of HRQOL [10]. Another multicentre follow-up study from China indicates that Covid-19 has a substantial effect on HRQOL, with some impacts persisting more than three months after discharge [11].

An HRQOL study using EQ-5D on a multi-ethnic Asian population in Singapore among Covid-19 and cardiovascular comorbid patients indicates that the mental health dimension of patient wellbeing was the most affected area [12]. An HRQOL study from Iran using the EQ-5D reports a significantly low HRQOL score among Covid-19 patients (0.6125) and indicates that socioeconomic factors (i.e., gender, age, educational status, employment status) and comorbidity status (i.e., having diabetes or cardiovascular disease) were significant predictors of HRQOL score [13].

Covid-19’s impact on HRQOL varies from country to country due to socioeconomic factors, the treatment modalities offered (and their outcomes) and variations in the disease’s severity and epidemiology [6]. However, although local evidence of the impact of Covid-19 on HRQOL is essential to inform national and regional Covid-19 treatment protocol designs, the disease’s impact on HRQOL in the Ethiopian or African context was unknown. Therefore, this study assessed the impact of Covid-19 and associated factors on HRQOL using primary data from confirmed cases in a Covid-19 treatment centre in South Central Ethiopia.

## Methods

### Study setting, design and population

This study employed a facility-based, cross-sectional study design. We conducted this study in the Arsi Zone at the Bokoji Hospital Covid-19 treatment centre, one of the largest Covid-19 treatment centres in South Central Ethiopia, which provides services for people from 28 districts and two town administrations.

**Fig. 1:**
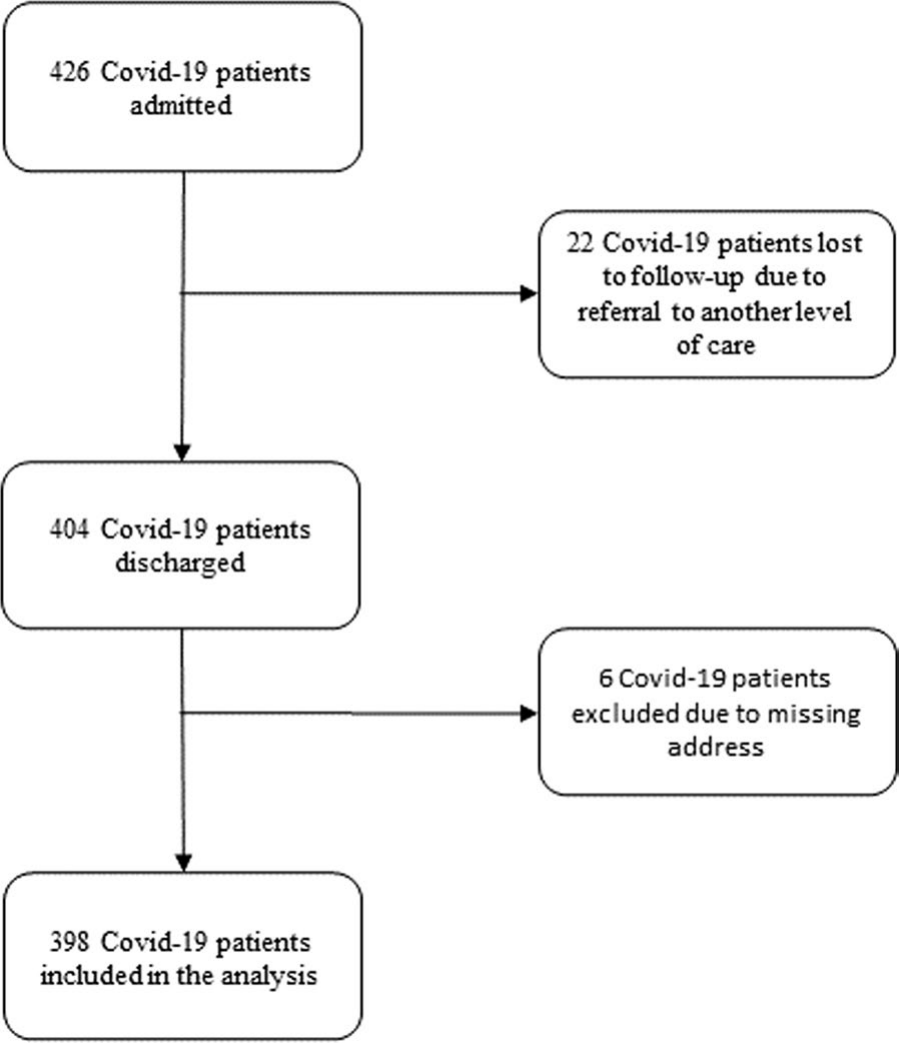
Flow diagram for study participants

The sample size was determined using single population formula with assumption type I error of 0.05, confidence interval 95%, proportion of good HRQOL (50%), and non-response 10%. The final calculated sample size was 422, and since the patients discharged and fulfilled the criteria were below this, all Covid-19 patients discharged from treatment were recruited for the study. The study population was all Covid-19 patients discharged from the treatment centre from July 1, 2020 through March 20, 2021. All Covid-19 patients discharged from the treatment centre after being cured or with consent for home-based care were included. Excluding all the Covid-19 patients referred to other treatment centre hospitals, incomplete medical records or deceased, 398 confirmed Covid-19 cases were included in the analysis (Fig. 1).

### Data collection and tools

To measure the HRQOL of Covid-19 patients, we employed the visual analogue scale (VAS) alongside the EQ-5D-3L questionnaire, which is the most common instrument for assessing HRQOL. The EQ-5D-3L includes five dimensions (mobility, self-care, usual activities, pain/discomfort, anxiety/depression), each with three levels to define possible health states (no problems, some problems, inability to/extreme problems). The VAS is a vertical graduated line (0–100) that indicates the overall health status of the respondent, 0 being the worst imaginable health state and 100 being the best imaginable. Four healthcare professionals collected the data after a two-day training on data collection procedures and the tools. Data collection was conducted using a face-to-face interview. Additionally, information on sociodemographic and clinical characteristics was extracted from patients’ medical records. The first author (AK) supervised the data collection.

### Study variables and operational definitions

The health state utility (HSU) was the dependent variable. In contrast, sociodemographic variables, like age, sex, marital status, residence, and clinical variables like general health status during admission, chronic illnesses, dexamethasone treatment, internasal oxygen use and the average length of stay were the independent variables. Patients general health status were defined as asymptomatic, mild, moderate, severe or critically ill, according to the WHO as well as Ethiopian national diagnosis and treatment protocol. ‘Asymptomatic infections’ were defined as the absence of clinical signs and symptoms with a positive nucleic acid test, whereas ‘Mild Covid-19 disease’ was defined as the presence of mild clinical signs and symptoms without respiratory distress and the absence of imaging manifestations of pneumonia. ‘Moderate disease’ was defined as the presence of clinical signs of pneumonia (fever, cough, dyspnoea, and fast breathing) but without symptoms of severe pneumonia, including SpO2 ≥ 90% on room air. Severe disease was defined as the presence of at least one of the three conditions: respiratory distress, a respiratory rate ≥ of 30 beats/min; oxygen saturation in resting-state ≤ 90%; or an arterial blood oxygen partial pressure/oxygen concentration ≤ 200 mmHg. Critically ill was defined as respiratory failure requiring mechanical ventilation, shock or combined organ failure requiring intensive care unit (ICU) monitoring and treatment [14, 15]. Health status at discharge was cured, transferred or discharged with consent. Cured was defined as the Covid-19 patients discharged after two times negative laboratory finding was confirmed. Discharged with consent was defined as Covid-19 patients discharged with consent after their one laboratory result was positive after at least 14 days stay in the treatment centre. Similarly, transfer was defined as Covid-19 patients transferred to other treatment centres for more management of Covid-19 or complications due to underlying diseases.

### Data analysis

The HRQOL results measured by the EQ-5D-3L tool were converted to a health state utility (HSU) using the Zimbabwe tariff value set, while the VAS scores were taken directly as another HSU (HSU-VAS) [16]. Both the HUI from the EQ-5D-3L and the overall HSU-VAS from the VAS score were analysed as a continuous variables. We used frequencies and percentages to summarise the sociodemographic and clinical characteristics of the participants and summarised the HUIs by median with interquartile range (IQR) and mean with a standard deviation (SD). We compared the average HUI and HSU-VAS across various groups of sociodemographic and clinical characteristics using the Mann–Whitney U test or the Kruskal–Wallis test. We examined the data for normality, multicollinearity, and heteroscedasticity statistical assumptions. To assess the factors associated with HSU simultaneously, we employed a multiple linear regression. We calculated coefficient (β) and 95% confidence intervals (CIs). A P-value of less than 0.05 was considered statistically significant. We used STATA version 15 for data analysis.

### Ethical approval

This study was approved by the Ethical Review Board of Arsi University College of Health Sciences. Informed consent was obtained from all the participants. We used the STROBE cross-sectional checklist when writing our report [17].

## Results

A total of 398 confirmed Covid-19 cases were included in the study. The average length of hospital stay was 14.3 days (SD: 4.78). The majority of the Covid-19 cases were male (60%), older than 55 years (28.9%) (Maximum = 95 years old) and residents of urban areas (61%). Regarding general health status on admission, 32.7% were severely ill, 20% had a moderate symptom, 23.4% had mild symptoms, and 23.9% were asymptomatic. Forty-five percent of the cases had some comorbidity, with diabetes mellitus (17.1%), hypertension (10.3%) and asthma (8.3%) being the top three comorbidities. Regarding the antibiotic treatment regimen, 37.2% were treated with azithromycin, while 32.9% received a combination of azithromycin and ceftriaxone. In addition, about one-third (29.1%) were treated with dexamethasone. Furthermore, nearly two-thirds (59.3%) received intranasal oxygen supplementation (Table 1).

**Table 1:** Demographic and clinical characteristics of Covid-19 patients admitted to a treatment centre in the Arsi Zone, 2020–2021

Demographic and clinical characteristics	Frequency (%)
Sex	
Female	159 (40.0)
Male	239 (60.0)
Age (mean = 41.5 (SD: 18.8)	
0–24 years	83 (20.9)
25–34	102 (25.6)
35–44	52 (13.1)
45–54	46 (11.6)
55 years and above	115 (28.9)
Residence	
Rural	156 (39.0)
Urban	242 (61.0)
Health status on admission	
Asymptomatic	95 (23.9)
Mild	93 (23.4)
Moderate	80 (20.0)
Severe	130 (32.7)
Comorbidity	
Yes	179 (45.0)
No	219 (55.0)
Type of comorbidity	
Diabetes mellitus	68 (17.1)
Hypertension	41 (10.3)
Asthma	33 (8.3)
Chronic pulmonary disease	30 (7.5)
Chronic cardiac diseases	23 (5.8)
Malignancy	11 (2.8)
Chronic kidney disease	7 (1.8)
HIV/AIDS	6 (1.5)
Types of antibiotic administered	
Azithromycin only	148 (37.2)
Azithromycin + ceftriaxone	131 (32.9)
Azithromycin + vancomycin + ceftazidime	50 (12.6)
Azithromycin + ceftriaxone + metronidazole	30 (7.5)
Azithromycin + ceftriaxone + vancomycin	24 (6.0)
Azithromycin + ceftriaxone + amoxicillin	13 (3.3)
Azithromycin + ceftriaxone + ceftazidime	2 (0.5)
Dexamethasone used	
Yes	116 (29.1)
No	282 (70.9)
Intranasal oxygen used	
Yes	162 (59.3)
No	236 (40.7)
Length of hospital stay (mean = 14.3 SD:4.8)	
1–7 days	12 (3.0)
8–14 days	248 (61.8)
15–21 days	113 (28.4)
22–28 days	13 (3.3)
More than 28 days	14 (5.5)

The overall mean HSU of the EQ-5D index score was 0.688 (SD: 0.285) (Table 2). The overall mean HSU of the VAS score was 0.690 (SD: 0.129) (Table 3). There was significant variation in the mean HSU score across age groups (*p* < 0.001). The mean EQ-5D index score among those older than 55 years was 0.567, while it was 0.783 among those younger than 25 years. In general, the mean EQ-5D index scores were significantly lower for respondents with comorbidity (0.574) than for those without comorbidity (0.777) (*p* < 0.001) (Table 3). The EQ-5D index score was significantly lower among those with hypertension, chronic cardiac diseases, chronic pulmonary disease, asthma, chronic kidney disease and diabetes mellitus than among those who did not have those comorbidities. Those who received dexamethasone and supplemental intranasal oxygen had significantly lower EQ-5D index scores than those who did not receive them (*p* < 0.001), but there was no difference in the EQ-5D index score across gender and place of residence (urban vs. rural). The mean HSU for VAS score was 0.629 among those older than 55 years, whereas it was 0.732 among those younger than 25 years. Moreover, the mean VAS scores were significantly lower for respondents on intranasal oxygen (0.604) than their counterparts (0.749) (*p* < 0.001). Respondents who received dexamethasone treatment (*p* < 0.001),with hypertension (*p* < 0.002), chronic cardiac disease (*p* < 0.005), chronic pulmonary disease (*p* < 0.001), diabetic mellitus (*p* < 0.001) and asthma (*p* < 0.001) were associated with lower VAS score (Table 3).

**Table 2:** Comparison of the HSU values of the EQ-5D-3L tool across the demographic and clinical characteristics of Covid-19 patients admitted to a treatment centre in the Arsi Zone, 2020–2021

Variable	Health utility value (EQ-5D-3L)					
	Median	IQR (P25, P75)		Mean	SD	*p* value
Sex						
Female	0.787	0.596	0.833	0.684	0.302	0.818
Male	0.787	0.596	0.854	0.689	0.274	
Age						
0–24	0.787	0.596	1.000	0.783	0.199	< 0.001
25–34	0.787	0.596	1.000	0.778	0.213	
35–44	0.787	0.596	0.787	0.649	0.328	
45–54	0.691	0.596	0.854	0.653	0.213	
55+	0.596	0.596	0.787	0.567	0.314	
Residence						
Rural	0.787	0.596	0.854	0.692	0.282	0.967
Urban	0.787	0.596	0.833	0.685	0.288	
Comorbidity						
No	0.787	0.596	1.000	0.777	0.257	< 0.001
Yes	0.596	0.596	0.787	0.574	0.279	
Hypertension						
No	0.787	0.596	0.854	0.699	0.285	0.001
Yes	0.596	0.596	0.787	0.580	0.267	
Chronic cardiac diseases						
No	0.787	0.596	0.854	0.697	0.280	0.004
Yes	0.596	0.596	0.787	0.518	0.320	
Chronic pulmonary disease						
No	0.787	0.596	0.854	0.703	0.277	< 0.001
Yes	0.596	0.596	0.596	0.499	0.311	
Asthma						
No	0.787	0.596	0.854	0.706	0.252	< 0.001
Yes	0.596	0.469	0.596	0.487	0.329	
Chronic kidney disease						
No	0.787	0.596	0.854	0.690	0.286	0.029
Yes	0.596	0.361	0.596	0.535	0.186	
Diabetes mellitus						
No	0.787	0.596	1.000	0.711	0.281	< 0.001
Yes	0.596	0.596	0.787	0.575	0.280	
Malignancy						
No	0.787	0.596	0.854	0.687	0.288	0.859
Yes	0.787	0.596	0.833	0.708	0.147	
HIV/AIDS						
No	0.787	0.596	0.843	0.688	0.285	0.354
Yes	0.692	0.596	0.787	0.607	0.270	
Dexamethasone used						
No	0.787	0.596	1.000	0.735	0.280	< 0.001
Yes	0.596	0.596	0.787	0.571	0.262	
Intranasal oxygen used						
No	0.787	0.787	1.000	0.816	0.180	< 0.001
Yes	0.596	0.596	0.596	0.500	0.305	
Length of hospital stay						
1–7 days	0.691	0.596	0.866	0.718	0.227	0.002
8–14 days	0.787	0.596	1.000	0.719	0.283	
15–21 days	0.596	0.596	0.787	0.622	0.297	
22–28 days	0.596	0.596	0.787	0.715	0.197	
More than 28 days	0.596	0.469	0.787	0.604	0.241	
Overall	0.787	0.596	0.833	0.688	0.285	

SD = standard deviation; IQR = interquartile range; P-values are from the Mann–Whitney U test or Kruskal–Wallis test

**Table 3:** Comparison of the HSU values of the VAS across the demographic and clinical characteristics of Covid-19 patients admitted to a treatment centre in the Arsi Zone, 2020–2021

Variable	Health utility value (VAS)					
	Median	IQR (P25, P75)		Mean	SD	*p* value
Sex						
Female	0.700	0.600	0.800	0.689	0.134	0.961
Male	0.700	0.600	0.800	0.692	0.127	
Age						
0–24	0.725	0.610	0.860	0.732	0.126	< 0.001
25–34	0.750	0.650	0.840	0.734	0.121	
35–44	0.700	0.580	0.780	0.686	0.126	
45–54	0.680	0.600	0.780	0.678	0.123	
55+	0.620	0.560	0.710	0.629	0.118	
Residence						
Rural	0.700	0.600	0.810	0.695	0.132	0.927
Urban	0.700	0.600	0.790	0.688	0.128	
Comorbidity						
No	0.750	0.640	0.850	0.738	0.129	< 0.001
Yes	0.620	0.570	0.710	0.632	0.103	
Hypertension						
No	0.700	0.600	0.800	0.697	0.131	0.002
Yes	0.610	0.580	0.700	0.634	0.096	
Chronic cardiac diseases						
No	0.700	0.600	0.800	0.695	0.129	0.005
Yes	0.630	0.570	0.700	0.613	0.102	
Chronic pulmonary disease						
No	0.700	0.600	0.800	0.697	0.130	< 0.001
Yes	0.605	0.570	0.660	0.606	0.081	
Asthma						
No	0.700	0.600	0.800	0.699	0.129	< 0.001
Yes	0.590	0.560	0.640	0.601	0.096	
Chronic kidney disease						
No	0.700	0.600	0.800	0.692	0.129	0.081
Yes	0.630	0.550	0.660	0.607	0.094	
Diabetes mellitus						
No	0.705	0.600	0.820	0.705	0.129	< 0.001
Yes	0.700	0.570	0.700	0.622	0.109	
Malignancy						
No	0.700	0.600	0.800	0.691	0.130	0.782
Yes	0.710	0.600	0.780	0.675	0.117	
HIV/AIDS						
No	0.700	0.600	0.800	0.691	0.129	0.531
Yes	0.665	0.590	0.750	0.653	0.112	
Dexamethasone used						
No	0.730	0.610	0.848	0.718	0.131	< 0.001
Yes	0.600	0.570	0.700	0.625	0.097	
Intranasal oxygen used						
No	0.750	0.695	0.850	0.749	0.116	< 0.001
Yes	0.600	0.560	0.660	0.604	0.096	
Length of hospital stay						
1–7 days	0.690	0.610	0.820	0.703	0.133	0.004
8–14 days	0.720	0.600	0.820	0.709	0.229	
15–21 days	0.640	0.590	0.730	0.657	0.122	
22–28 days	0.640	0.600	0.750	0.687	0.127	
More than 28 days	0.615	0.530	0.730	0.629	0.131	
Overall	0.700	0.600	0.800	0.690	0.129	

SD = standard deviation; IQR = interquartile range; P-values are from the Mann–Whitney U test or Kruskal–Wallis test

The multiple linear regression analysis results are presented in Table 4. The patient’s age, having asthma as comorbidity, and general health status during admission were significantly associated with low HSU values. On the other hand, those who were treated with dexamethasone had significantly higher HSU values (P-value < 0.05) (Table 4).

**Table 4:** Multiple linear regression analysis for factors associated with HSU values of Covid-19 patients admitted to a treatment centre in the Arsi Zone, 2020–2021

Variables	HSU values of the EQ-5D (Adjusted R^2^: 45%)				HSU values of the VAS (Adjusted R^2^: 55%)			
	Coef	*p* value	[95% CI]		Coef	*p* value	[95% CI]	
Sex (Ref: Female)	0.024	0.276	− 0.019	0.068	0.013	0.155	− 0.005	0.031
Age (in year)	− 0.001	0.048	− 0.002	0.000	0.000	0.030	− 0.001	0.000
Residence (Ref: Rural)	− 0.003	0.905	− 0.047	0.042	− 0.004	0.695	− 0.022	0.014
Hypertension (Ref: No)	− 0.017	0.652	− 0.089	0.056	− 0.015	0.326	− 0.044	0.015
Chronic cardiac diseases (Ref: No)	− 0.032	0.512	− 0.129	0.065	− 0.018	0.371	− 0.058	0.022
Chronic pulmonary disease (Ref: No)	− 0.018	0.678	− 0.101	0.066	− 0.007	0.689	− 0.041	0.027
Asthma (Ref: No)	− 0.091	0.024	− 0.169	− 0.012	− 0.036	0.029	− 0.068	− 0.004
Chronic kidney disease (Ref: No)	0.022	0.788	− 0.140	0.185	− 0.003	0.933	− 0.069	0.064
Diabetes mellitus (Ref: No)	− 0.008	0.791	− 0.069	0.053	− 0.017	0.192	− 0.041	0.008
Malignance (Ref: No)	− 0.009	0.887	− 0.140	0.121	− 0.038	0.158	− 0.092	0.015
AIDS HIV (Ref: No)	0.039	0.664	− 0.137	0.215	0.030	0.409	− 0.042	0.103
Dexamethasone use (Ref: No)	0.089	0.002	0.033	0.145	0.026	0.026	0.003	0.049
Intra nasal oxygen use (Ref: No)	− 0.042	0.251	− 0.114	0.030	0.012	0.421	− 0.017	0.042
*Health status on admission*								
Mild (Ref: No symptom)	− 0.093	0.004	− 0.156	− 0.031	− 0.064	0.000	− 0.089	− 0.038
Moderate (Ref: No symptom)	− 0.269	0.000	− 0.341	− 0.197	− 0.171	0.000	− 0.200	− 0.142
Severe/ critical (Ref: No symptom)	− 0.445	0.000	− 0.537	− 0.353	− 0.243	0.000	− 0.281	− 0.206
Length of stay (in days)	− 0.001	0.767	− 0.005	0.004	− 0.001	0.237	− 0.003	0.001
_cons	0.955	0.000	0.870	1.039	0.847	0.000	0.812	0.881

Coef: Coefficient; CI: Confidence Interval; SE: Standard Error; Ref: Reference category

## Discussion

Covid-19 has caused significant psychological and physiological stress to patients and their families worldwide. This study examined the HRQOL of Covid-19 patients using the EQ-5D-3L and VAS tools. The overall mean VAS score was 0.690 (median = 0.700) in our study. This was similar with study from Egypt (72.2) [20],Peru (76) [21],Spain (66.36) [13], China (85.52) [20] and Addis Ababa, Ethiopia (69.44) [22]. Moreover, the mean EQ-5D index score among Covid-19 patients on discharge was 0.688 (SD = 0.285).In general, these findings are in line with those of a study in Iran that reports an EQ-5D index score of 0.612 [13] and a Belgian study with an EQ-5D index score of 0.620 [18], but our findings are substantially lower than those of studies from Norway (EQ-5D index score: 0.820) [19], China (EQ-5D index score: 0.949) and Hong Kong (EQ-5D index score: 0.897) [20, 21]. Variations in the HRQOL evaluation method employed (i.e., health utility tariff, tools, scale, study participant sampling) may also, to some extent, contribute to the discrepancy. The studies in China, Iran, Argentina, Belgium and Norway employed the EQ-5D-5L instrument, while our study employed the EQ-5D-3L. Moreover, the variation in age distribution may be a driver of variation in HRQOL across countries, and the population in our study was relatively younger (mean age = 40) than in other places.

In our study, respondents age 55 and above years old had a significantly lower HRQOL than younger people (0.567 vs 0.783). This is similar with finding from, Iran(0.554 vs 0.618) [13], China (0.963 vs 0.889) [20], and South Africa (0.655 vs 0.501) [22]. Moreover, in regression analysis, age was also significantly associated with health utility status. This finding is in line with a find from Argentina study [23]. According to the Argentian study, those older than 50 were 5.6 times more likely to have poor HRQOL than their counterparts. This finding can be explained by increased mental stress, comorbidity and debilitation in the physical condition of older people [24]. In contrast, those middle-aged males (26–35 years) patients were five times more at risk of having poor HRQOL in Saudi Arabia compared with older counterparts (55–65 years) [25].

According to our study, comorbidity, especially asthma (Table 4), is significantly associated with lower health utility scores (Table 2). This similar with studies from Vietnam [26], Palestine [27], Peru [28], India [29] and Addis Ababa, Ethiopia [30]. The mean VASscores were significantly lower for respondents with comorbidity (62) than for those without it (75) (*p* < 0.001). In general, comorbidities (such as hypertension, chronic cardiac diseases, chronic pulmonary disease, asthma, chronic kidney disease and diabetes mellitus) were significantly associated with low HSU VAS scores. Studies from Vietnam (70.8 vs 63.3) [31], China (97.9 Vs 82.8) [20] and Palestine (80 vs 70) [27] revealed that individuals with chronic diseases have a lower HRQOL than those without comorbid disease, perhaps because those with comorbidities develop anxiety or depression in response to misinformation disseminated about the impact of the virus in these communities [25, 32].

We found that Covid-19 patients who received dexamethasone and intranasal oxygen supplementation had lower EQ-5D index scores than those who did not receive them (*p* < 0.001), perhaps because those who needed those treatments had a severe form of the illness. Furthermore, those with a length of stay (LOS) of more than 15 days in hospital had lower EQ-5D index scores than their counterparts. Studies from China, Spain and Argentina also revealed that increased LOS is associated with poor HRQOL [10, 33–35]. This poor HRQOL might be due to confinement to one place, increasing anxiety and reducing the HRQOL in general.

This study represents the first comprehensive analysis of the HRQOL of Covid-19 patients in the Ethiopian setting to the best of our knowledge. We conducted the study in a setting that accommodated patients from 28 districts. However, our study has some limitations. First, because the study collected HRQOL data based on patient preferences, the patients might over or underestimated their health status during the interview. Second, we have no HRQOL estimate for 22 patients who lost to follow-up due to referral to another level of care. In addition, this study used the Zimbabwe tariff due to the lack of an Ethiopian tariff, and this limitation could impact the estimation of the real Ethiopian HRQOL against the disease, as there are many differences between the two countries. Moreover, due to the study’s cross-sectional design, we could not compare the HRQOL of patients before the Covid-19 infection.

## Conclusion

In conclusion, the Covid-19 disease substantially impaired the HRQOL of patients in Ethiopia. Elderly patients and Covid-19 patients with comorbidity had notably low HRQOLs. Therefore, close clinical follow-up and psychological treatment should be encouraged for these groups. Moreover, the health utility values from this study can be used to evaluate quality adjusted life years for future cost-effectiveness analyses of prevention and treatment interventions against Covid-19.

## Data Availability

The data sets used or analysed in this study are available from the corresponding author upon reasonable request.

## Abbreviations

EQ-5D-3L: Euro Qal–5 Dimension–3 Level
HRQOL: Health-related quality of life
HIV: Human immune virus
HUI: Health utility index
HSU: Health state utility
LOS: Length of stay
ICU: Intensive care unit
SD: Standard deviation
SF-36: Standard format–36
VAS: Visual analogue scale
WHO: World Health Organization
